# Cognitive Decline and Retirement: Findings From the Canadian Longitudinal Study on Aging

**DOI:** 10.1093/geroni/igab046.3472

**Published:** 2021-12-17

**Authors:** Catherine Gosselin, Meghan Désilets-Jutras, Benjamin Boller

**Affiliations:** 1 Université du Québec à Trois-Rivières, Trois-Rivières, Quebec, Canada; 2 Université du Quebec à Trois-Rivières, Université du Québec à Trois-Rivières, Quebec, Canada; 3 Université du Québec à Trois-Rivières, Université du Québec à Trois-Rivières, Quebec, Canada

## Abstract

Since increasing life expectancy leads to a longer period of retirement, several studies have been investigating the possible impact of retirement on cognitive health. Several epidemiological studies with cross-sectional designs have reported a negative association between retirement and cognitive capacities. However, very few studies with longitudinal designs have confirmed the negative effect of retirement on cognitive functioning. The present study was conducted to investigate the impact of retirement on cognitive capacities among older Canadians. We used data from the Comprehensive cohort of the Canadian Longitudinal Study on Aging (CLSA) to compare performance retirees and workers (N = 1442), 45 to 85 years of age at baseline. Memory and executive functioning were assessed using standardized assessment tools at baseline and at three-year follow up. Retirees and workers were matched for age, gender and education using the nearest neighbor propensity score method with a caliper of 0.02. Mixed ANOVA and post hoc analyses were conducted separately for the English- and French-speaking samples. Results for the English-speaking sample showed a significant decline on both the Stroop and the Mental Alternation Task for retirees compared to workers from baseline to follow-up. These results support previous cross-sectional studies that have demonstrated a negative effect of retirement on executive functioning. The absence of significant results in the French-speaking sample will be discussed in terms of sample size and professional occupation.

